# Piezo1 Activates an Autocrine Angiopoietin-2-Integrin Signaling Loop in Schlemm’s Canal to Regulate Intraocular Pressure

**DOI:** 10.1101/2025.10.24.683742

**Published:** 2025-10-24

**Authors:** Naoki Kiyota, Dilip K. Deb, Benjamin R. Thomson, Susan E. Quaggin

**Affiliations:** 1 Feinberg Cardiovascular and Renal Research Institute, Northwestern University Feinberg School of Medicine, Chicago.; 2 Division of Nephrology and Hypertension, Northwestern University Feinberg School of Medicine, Chicago, IL.; 3 Department of Ophthalmology, Northwestern University Feinberg School of Medicine, Chicago, IL.; 4 Department of Biomedical Engineering, Northwestern University McCormick School of Engineering, Evanston, IL.

**Keywords:** angiopoietin-2, PIEZO1, integrin α9β1, Schlemm’s canal, mechanotransduction, intraocular pressure, glaucoma

## Abstract

Intraocular pressure (IOP) is the most important risk factor in glaucoma pathogenesis. IOP is regulated by segmental, flow-dependent responses of the inner-wall endothelium of Schlemm’s canal (SC), and dysfunction of this system contributes to ocular hypertension and glaucoma. Here, we identify a cell-autonomous PIEZO1–ANGPT2–integrin α9 (ITGA9)–focal adhesion kinase (FAK) mechanotransduction pathway that enables SC endothelial cells to respond to pressure and flow stimuli to maintain IOP homeostasis. Pharmacological activation of the mechanosensitive ion channel PIEZO1 with Yoda1 enhanced ITGA9 accumulation at cell–cell junctions in vitro and induced FAK phosphorylation in an ITGA9- and ANGPT2-dependent manner. In vivo in SC, PIEZO1 activation reduced intracellular ANGPT2, increased junctional ITGA9, and was associated with phosphorylation of FAK and the ANGPT receptor TIE2 (encoded by *TEK*). Endothelial-specific deletion of *Piezo1* or inducible knockout of *Itga9* in mice resulted in SC narrowing, elevated IOP, and peripheral retinal ganglion cell loss; double heterozygotes exhibited more severe phenotypes than either single heterozygote, consistent with functional interaction within a single pathway. Moreover, in eyes from *Piezo1* and *Itga9* mutants, endothelial cell proliferation was reduced, and similar findings have been reported in *Angpt2* mutants, supporting a model in which the autocrine flow–PIEZO1–ANGPT2–ITGA9 axis links mechanical stimuli to structural adaptation of SC. These findings suggest that therapeutically targeting this pathway may restore outflow and prevent glaucoma.

## Introduction

Glaucoma is a leading cause of blindness affecting ~64 million people worldwide [1]. While several distinct forms of glaucoma exist (reviewed in [2]), loss of retinal ganglion cells (RGCs) and deformation of the optic nerve are directly responsible for vision loss in all forms of the disease. Elevated intraocular pressure (IOP) due to increased aqueous humor outflow resistance is the primary risk factor for glaucoma pathogenesis, and lowering of IOP slows vision loss in both high-pressure and normotensive glaucoma [3,4]. Schlemm’s canal (SC), a large vessel in the ocular anterior segment, is responsible for the majority of aqueous humor outflow, and alterations in physical characteristics of SC endothelial cells are associated with increased outflow resistance and elevated IOP, making them a key target for glaucoma therapy [5,6].

SC is a unique endothelium, expressing a hybrid phenotype with both venous and lymphatic characteristics [7,8]. Consistent with its central role in modulating IOP and aqueous humor outflow, many genes regulating SC development and function have been linked with glaucoma in patients or in animal models. Some of the best characterized belong to the angiopoietin (ANGPT) signaling pathway, comprised of the ANGPT ligands and the SC-expressed receptor TIE2 (encoded by *Tek*) [9,10]. Loss of function variants in TEK, or its trabecular meshwork (TM)-expressed ligand ANGPT1 cause primary congenital glaucoma (PCG), a severe form of pediatric glaucoma, in humans and mice and are associated with increased risk of primary open angle glaucoma (POAG) in adults [9–11]. In addition to this well-understood paracrine signaling pathway, a second ANGPT ligand, ANGPT2, is expressed by endothelial cells of SC itself and acts in an autocrine fashion [10]. In mice, knockout studies have suggested that this pathway is less important than paracrine ANGPT1 signaling for SC development, but ANGPT2 variants are associated with glaucoma in humans and a variant in the 3’UTR of ANGPT2 that increases ANGPT2 expression leads to increased SC size in mice and is associated with reduced POAG risk in humans, suggesting that ANGPT2 has a significant functional role [12].

Furthermore, loss of both Angpt1 and Angpt2 led to a more severe SC phenotype than loss of Angpt1 alone, indicating compensation by Angpt2 [10]. In addition, ANGPT2 is known to have TIE2 independent roles in other vascular beds, functioning through integrins to regulate endothelial permeability, migration and cell proliferation [13]. In lymphatic endothelial cells (LECs), which share key features with SC, we recently showed that autocrine ANGPT2–TIE2 signaling is modulated by the mechanosensitive Ca^2+^ channel PIEZO1, enabling LECs to transduce shear stress into TIE2-dependent molecular and functional responses [14,15]. Like lymphatic vessels, SC is regulated by mechanical stimuli and shear stress [16]. Given that *Piezo1* regulates IOP in mice and *PIEZO1* variants associate with primary open-angle glaucoma (POAG), we speculated that it plays a similar role in SC [17,18]. Here, we interrogated this pathway in detail, showing that, indeed, this pathway is present in SC. Moreover, we also discovered that in addition to Tie2 phosphorylation and downstream signaling, PIEZO1-induced ANGPT2 secretion regulates integrin signaling through the endothelial integrin α9β1 and phosphorylation of focal adhesion kinase (FAK). This new signaling cascade is critical for SC function, and deletion of *Itga9* or endothelial deletion of *Piezo1* lead to increased IOP in mice.

## Results

### PIEZO1 activation promotes ANGPT2 secretion and triggers downstream TIE2 signaling in SC.

**Figure 1.**
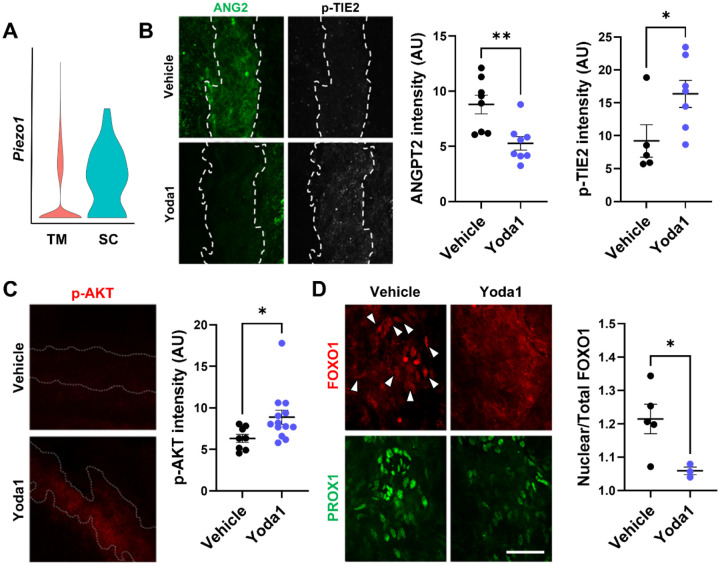
PIEZO1 activation in Schlemm’s canal (SC) acutely mobilizes ANGPT2 and engages AKT–FOXO1 signaling in vivo. (A) Violin plot of Piezo1 expression in single-cell RNA-seq showing enrichment in SC endothelium (blue) versus trabecular meshwork (TM, red). (B) ANGPT2/p-TIE2 immunostaining on limbal SC whole-mounts 30 min after intracameral injection (PBS control vs Yoda1, 20 μM in PBS, 1 μL at 100 nL/min; unilateral, contralateral eye PBS); left, ANGPT2 (green); right, p-TIE2 (white/greyscale); dashed lines outline SC; Yoda1 reduces intracellular ANGPT2 and concomitantly increases p-TIE2 in the same regions. (C) p-AKT immunostaining (red) is elevated in Yoda1-treated SC. (D) Co-immunostaining of FOXO1 (red) and PROX1 (green). White arrowheads indicate PROX1+ nuclei with prominent nuclear FOXO1 signal in vehicle-treated SC endothelium, which is reduced after Yoda1. Right, quantification of the FOXO1 nuclear-to-total intensity ratio in PROX1+ cells. All images acquired with identical settings; unpaired two-tailed Student’s t-test for quantifications. Scale bars: (B–C) 100 μm; (D) 50 μm.

We previously reported that activation of PIEZO1 in primary cultured LECs initiates a mechanotransduction cascade involving ANGPT2 secretion, TIE2 phosphorylation, and nuclear export of the transcription factor FOXO1, ultimately upregulating lymphatic valve–associated genes [14]. Although SC endothelial cells exhibit aspects of lymphatic endothelial fate, they are distinct from true lymphatics and retain features of their original blood-vascular phenotype [7,8]. To examine whether a similar PIEZO1-dependent ANGPT2–TIE2 signaling axis exists in SC, we first analyzed published single-cell RNA-seq datasets [19] and confirmed PIEZO1 expression in SC endothelial cells. As reported, PIEZO1 was present in the trabecular meshwork (TM), but we observed even higher expression in SC endothelium (Fig. 1A). Next, to determine whether this autocrine PIEZO1–ANGPT2 pathway regulates TIE2 signaling in SC as it does in lymphatics, we activated PIEZO1 channels in SC endothelium using the small-molecule agonist Yoda1. Yoda1 (20 μM in PBS containing 0.5% DMSO, 1 μL; unilateral) was delivered by intracameral injection at 100 nL/min; 30 min later, eyes were enucleated, SC whole mounts were prepared, and samples were imaged by confocal microscopy. The contralateral eye received vehicle (PBS + 0.5% DMSO) and served as the control. Yoda1 treatment led to reduced intracellular ANGPT2 staining, indicating secretion, together with a concomitant increase in phosphorylated TIE2 (p-TIE2) within the same SC regions (Fig. 1B; unpaired two-tailed t-test, n = 6–8 eyes per group).

As in LECs in vitro, this acute ANGPT2 release was accompanied by activation of downstream pathways, as indicated by elevated p-AKT (Fig. 1C; unpaired two-tailed t-test, n = 8–13 eyes per group, *P* = 0.0401) and a decrease in the FOXO1 nuclear/total intensity ratio in SC endothelial cells (Yoda1: 1.059 ± 0.011 vs vehicle: 1.214 ± 0.044; unpaired two-tailed t-test, n = 3–5 eyes per group, *P* = 0.022; Fig. 1D).

### PIEZO1-induced ANGPT2 secretion drives FAK phosphorylation via an ITGA9-dependent cascade

**Figure 2.**
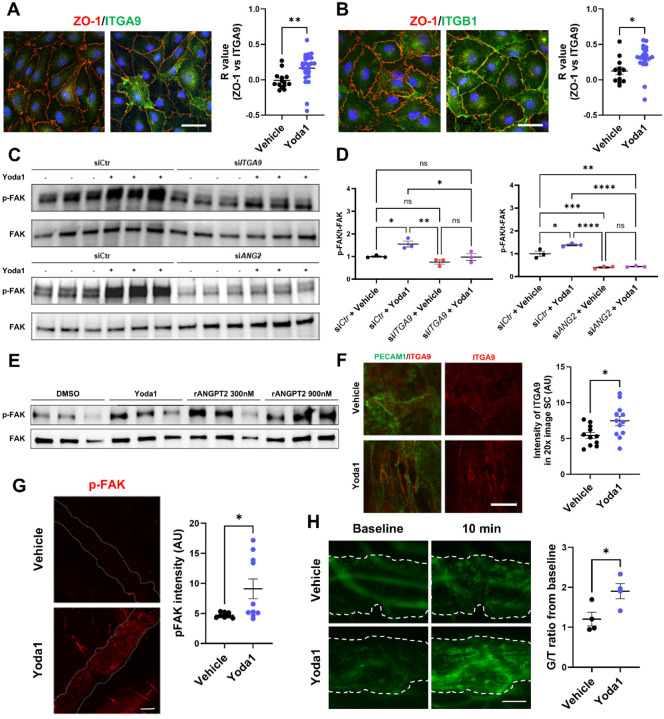
PIEZO1-induced *ANGPT2*–α9β1 signaling clusters integrins and activates FAK in vitro and in vivo. (A) HDLECs treated with DMSO or Yoda1 (2 μM, 30 min) stained for ITGA9 (green) and ZO-1 (red) show increased junctional co-localization; Pearson’s correlation coefficient (R) was computed with ImageJ Coloc2. (B) Under the same conditions, ITGB1 (green) and ZO-1 (red) co-localization likewise increases. (C) Immunoblots of p-FAK and total FAK from HDLECs ±Yoda1 with siCtrl vs si*ITGA9* show Yoda1-induced p-FAK that is attenuated by *ITGA9* knockdown. (D) Parallel blots for siCtrl vs si*ANGPT2* demonstrate loss of Yoda1-induced p-FAK with *ANGPT2* knockdown. (E) Recombinant *ANGPT2* alone elevates p-FAK without altering total FAK. (F) In vivo, limbal SC whole-mounts 30 min after intracameral PBS (control) or Yoda1 (20 μM in PBS, 1 μL at 100 nL/min; unilateral, contralateral PBS) stained for PECAM1 (green) and ITGA9 (red) show increased ITGA9 intensity with Yoda1. (G) p-FAK immunostaining in SC is higher after Yoda1 than PBS. (H) Ex vivo Ca^2+^ imaging in *Cdh5*-CreERT2; Salsa6f limbal strips (G/R ratio) shows a greater Yoda1-evoked increase versus vehicle. Dashed lines delineate SC; images were acquired with identical settings. Statistics: two-group comparisons used unpaired two-tailed Student’s t-test; multi-group comparisons used one-way ANOVA with Tukey–Kramer’s post hoc test. Scale bars: (A,B) 50 μm; (F) 25 μm; (G,H) 100 μm. Data are presented as mean ± SE. *P < 0.05, **P < 0.01, ns = not significant.

In addition to its established role as a TIE2 ligand, ANGPT2 can regulate angiogenesis and endothelial behavior through integrin engagement, independent of TIE2 [13,20,21]. Integrins reside constitutively at the plasma membrane but undergo activation-dependent conformational changes and lateral clustering at cell–cell or cell–matrix contacts, where they nucleate focal adhesions and downstream signaling [22,23]. Among endothelial integrins, α9β1 is a canonical lymphatic integrin essential for lymphatic-valve morphogenesis [24] and is reported in SC endothelium by transcriptomic profiling [8,25]. Moreover, the extracellular-matrix protein SVEP1 (Polydom) is a ligand for integrin α9β1 [26] and also binds TIE1 and ANGPT2 within the ANGPT–TIE signaling axis [27]. To test whether PIEZO1 modulates ITGA9 localization and downstream signaling via ANGPT2, we treated LECs with Yoda1. Yoda1 enhanced ITGA9 accumulation at cell–cell junctions, evidenced by increased colocalization with ZO-1 (Pearson’s R: 0.165 ± 0.041 vs DMSO −0.009 ± 0.032, P = 0.0073; Fig.2A). Because ITGA9 functions as a heterodimer with ITGB1, ITGB1/ZO-1 colocalization similarly increased (0.306 ± 0.042 vs 0.126 ± 0.052, P = 0.0130; Fig.2B), indicating junctional clustering of integrins. We next examined focal adhesion kinase (FAK), a canonical integrin effector: Yoda1 increased p-FAK without altering total FAK and this effect was attenuated by ITGA9 or ANGPT2 knockdown, indicating that PIEZO1-induced FAK phosphorylation requires ITGA9 and ANGPT2 (Fig. 2C, D). In LECs, recombinant ANGPT2 alone was sufficient to elevate p-FAK (Fig. 2E).

To determine whether PIEZO1 engages the same pathway in vivo in SC, we injected Yoda1 (20 μM in PBS containing 0.5% DMSO, 1 μL; unilateral) into the anterior chamber at 100 nL/min and analyzed SC 30 min later. ITGA9 signal intensity in SC endothelium increased significantly (Yoda1 7.47 ± 0.66 vs vehicle 5.44 ± 0.42; unpaired two-tailed t-test, n = 11–12 eyes per group, *P* = 0.0179; Fig. 2F), consistent with junctional clustering, and pFAK intensity rose correspondingly (Fig. 2G; unpaired two-tailed t-test, n = 9–10 eyes per group, *P* = 0.0233). To assess whether PIEZO1 activation produced Ca2+ influx, we generated Cdh5-CreERT2; Salsa6f mice and induced recombination with tamoxifen. Eyes were dissected, a limbal strip containing SC was mounted, and either vehicle (PBS + 0.5% DMSO) or Yoda1 (20 μM) was applied. Time-lapse imaging of the green/tdTomato (G/R) ratio in SC endothelial cells showed a greater increase with Yoda1 (Fig. 2H; n = 4 strips per group). Together, these findings show that PIEZO1 activation promotes junctional enrichment of integrin α9β1 and integrin-dependent FAK signaling in vitro, and engages parallel autocrine pathways—ANGPT2–TIE2 and ANGPT2–α9β1–FAK—in SC in vivo.

### *Piezo1* deletion narrows SC, elevates IOP, and reduces distal RGC density

**Figure 3.**
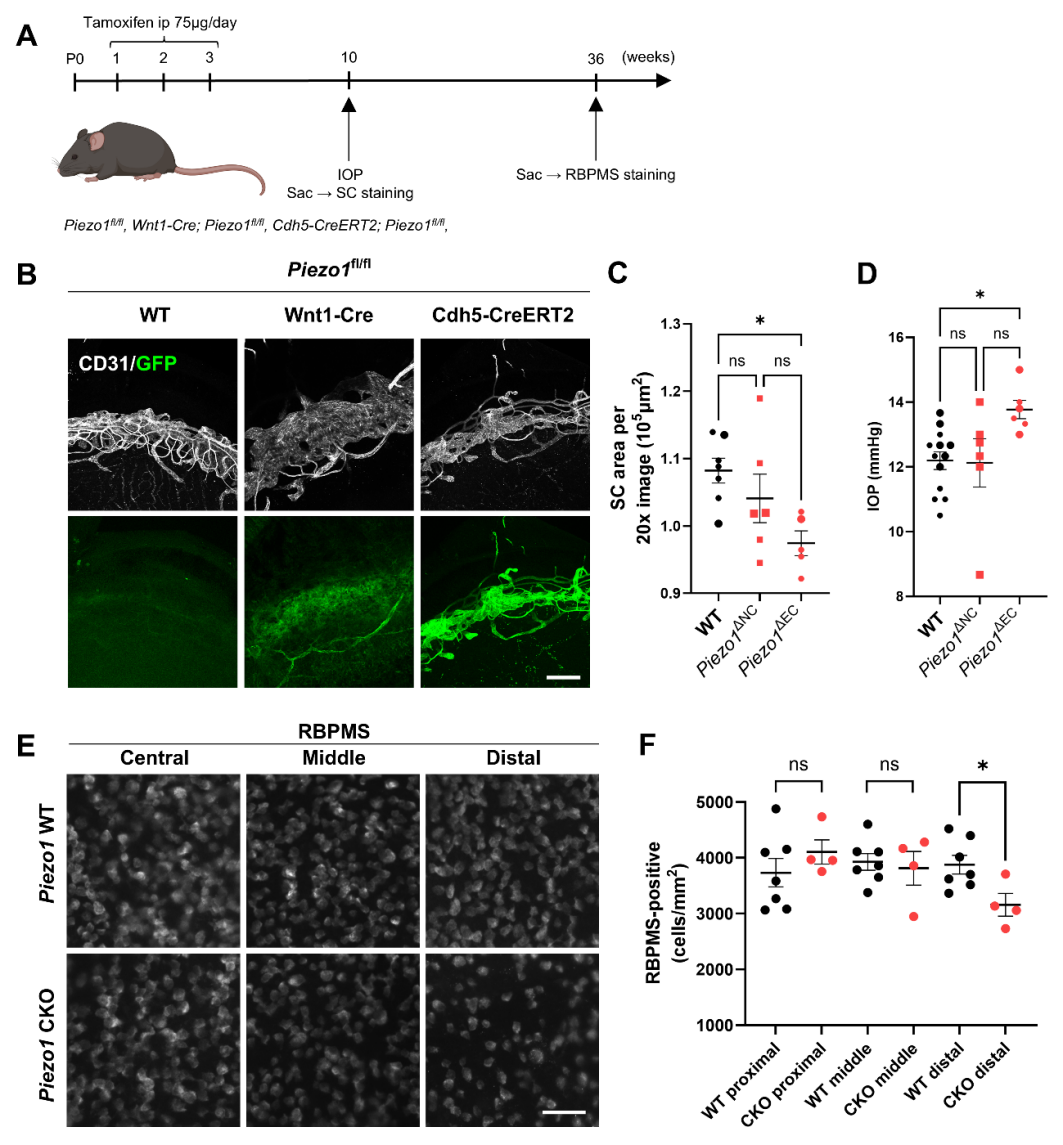
Endothelial-specific deletion of *Piezo1* elevates IOP and induces distal RGC loss. (A) Breeding/induction scheme for TM-specific (*Wnt1*-Cre) and endothelial-specific (*Cdh5*-CreERT2) deletion; tamoxifen 75 μg/day i.p. at P1–P3 for the endothelial model. (B) Recombination-site validation in limbal preparations showing CD31 (white) and mTmG-GFP (green), with TM labeling in the *Wnt1*-Cre line and SC-endothelial labeling in the *Cdh5*-CreERT2 line. (C) Representative limbal images of the SC region (CD31/GFP) across WT, *Piezo1*^ΔNC^, and *Piezo1*^ΔEC^. (D) IOP at 10 weeks (scatter) is increased in *Piezo1*^ΔEC^ versus WT and *Piezo1*^ΔNC^. (E) RBPMS-stained retinal flat mounts at 36 weeks (central, middle, distal) from WT and *Piezo1*^ΔEC^. (F) Quantification shows a selective reduction of RGC density in distal retina of *Piezo1*^ΔEC^, with central and middle regions unchanged. Statistics: two-group comparisons, unpaired two-tailed Student’s t-test; multi-group comparisons, one-way ANOVA with Tukey–Kramer post hoc test. Scale bars: (B) 100 μm; (E) 50 μm. Data are presented as mean ± SE. *P < 0.05, **P < 0.01, ns = not significant.

To define the functional role of PIEZO1 in SC in vivo, we used tissue-specific *Piezo1* knockout models. Prior studies detected *Piezo1* transcripts in the TM and, by single-cell profiling, in endothelial populations of the conventional outflow pathway, including SC [17,25]. Our single-cell RNA-seq analysis [19] likewise showed *Piezo1* expression in both TM and SC, with higher levels in SC endothelium (Fig. 1A), suggesting an SC-specific requirement. We generated two lines by crossing *Piezo1*^fl/fl^ with *Wnt1*-Cre (targeting neural crest–derived TM cells) or *Cdh5*-CreERT2 (targeting vascular endothelium, including SC). Hereafter, we refer to Wnt1-Cre–mediated deletion as neural-crest–specific deletion (ΔNC) and to Cdh5-CreERT2–mediated deletion as endothelial-specific deletion (ΔEC). Tamoxifen (75 μg/day, i.p., P1–P3) was administered to induce recombination in *Cdh5*-CreERT2 mice (Fig. 3A). Spatial specificity was validated by crossing each line to *Rosa26*^mTmG^ reporters, confirming efficient targeting of TM by *Wnt1*-Cre and of SC endothelium by *Cdh5*-CreERT2 (Fig. 3B). At 10 weeks of age, we measured IOP and then performed SC morphometry by CD31 immunostaining. *Piezo1*^ΔEC^ mice showed a significant reduction in SC area relative to controls, whereas *Piezo1*^ΔNC^ did not differ (Fig. 3C; one-way ANOVA with Tukey–Kramer, n = 5–7 eyes per group; adjusted *P* values as indicated), indicating that endothelial *Piezo1* is required to maintain SC area. Consistent with this phenotype, IOP at 10 weeks was higher in *Piezo1*^ΔEC^ than WT (13.77 ± 0.28 vs 12.78 ± 0.25 mmHg; Tukey-adjusted *P* = 0.026), while *Piezo1*^ΔNC^ did not differ from WT (12.12 ± 0.75 mmHg; Tukey-adjusted *P* = 0.438) (Fig. 3D; n = 6–13 mice per group).

To assess the consequences of chronic IOP elevation, we quantified RBPMS-positive RGCs—RBPMS is a pan-RGC marker—at 36 weeks in *Piezo1*^fl/fl^; *Cdh5*-CreERT2^−^ (control) and *Piezo1*^fl/fl^; *Cdh5*-CreERT2^+^ (*Piezo1*^ΔEC^) mice. Counts in the central, mid-peripheral, and distal retina revealed a selective reduction in the distal region of Piezo1^ΔEC^ mice (Fig. 3E,F; unpaired two-tailed t-tests within each eccentricity; n = 4–7 eyes per group; distal: P < 0.05), consistent with regionally biased RGC degeneration secondary to ocular hypertension. Image acquisition and quantification were performed using identical settings, with investigators blinded to genotype.

### *Itga9* deficiency impairs SC development and maintenance, leading to elevated intraocular pressure and RGC loss

**Figure 4.**
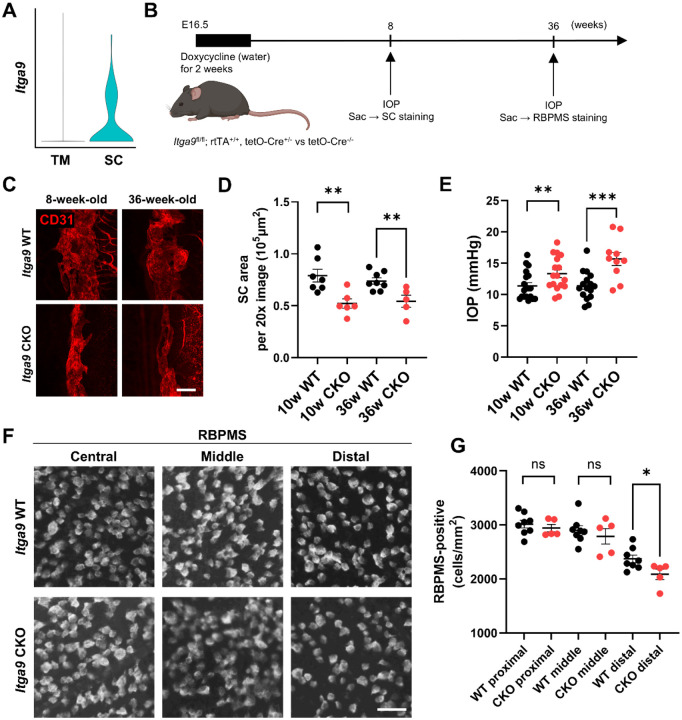
*Itga9* is required for SC development and IOP homeostasis (A) Violin plot showing *Itga9* expression levels derived from single-cell RNA sequencing of anterior segment tissues, demonstrating selective expression in Schlemm’s canal (SC) endothelial cells compared with trabecular meshwork (TM). (B) Schematic of the experimental timeline for doxycycline-inducible *Itga9* deletion. Doxycycline was administered from embryonic day 16.5 (E16.5) for 2 weeks to induce Cre expression in *Itga9*^fl/fl^; rtTA^+/+^; tetO-Cre^+/−^ (*Itga9* CKO) mice. (C) Representative immunofluorescence images of SC whole mounts at 8 weeks and 9 months, stained with anti-CD31 antibody to delineate SC morphology. (D) Quantification of SC area in Fig. 4C showing a significant reduction in *Itga9* CKO mice at both 8 weeks and 9 months compared with age-matched wild-type (WT) littermates. (E) Intraocular pressure (IOP) measured by rebound tonometry is significantly elevated in *Itga9* CKO mice at both 10 and 36 weeks of age. (F) Representative images of retinal flat mounts immunostained for RBPMS to label retinal ganglion cells (RGCs) from WT and *Itga9* CKO mice at 36 weeks, shown for central, middle, and distal regions. (G) Quantification of RBPMS-positive RGCs from Fig. 4F reveals a significant decrease in RGC density in the distal retina of *Itga9* CKO mice, with no difference in the central or middle regions. Statistical analyses were performed using two-tailed unpaired Student’s t-test (D, E, G). Data are presented as mean ± SE. *P < 0.05, **P < 0.01, ***P < 0.001, ns = not significant. Scale bars: (C) 100 μm; (F) 50 μm.

Our single-cell RNA sequencing analysis [19] revealed that Itga9 is predominantly expressed in SC endothelial cells, with minimal expression in TM cells (Fig.4A), consistent with prior reports that SC endothelium expresses integrin α9 [8]. While endothelial Itgb1 (β1 integrin) deletion has been reported to impair SC morphogenesis [28], the functional relevance of Itga9 in SC formation or maintenance has not been investigated. We generated a doxycycline-inducible conditional knockout (*Itga9*^fl/fl^; rtTA^+/+^; tetO-Cre^+/−^; hereafter *Itga9* CKO). Doxycycline was administered from embryonic day 16.5 (E16.5) for two weeks to induce *Itga9* deletion during fetal development (Fig. 4B). Cre-negative littermates (*Itga9*^fl/fl^; rtTA^+/+^; tetO-Cre^−^) served as controls. CD31 immunostaining at 8 weeks and 36 weeks (9 months) revealed a significant reduction of SC area in *Itga9* CKO at both ages (Fig. 4C,D; unpaired two-tailed t-tests within age, n = 5–8 eyes per group; 8 w: *P* < 0.01; 36 w: *P* < 0.05). Consistently, IOP was higher in CKO than controls at 10 weeks and 36 weeks (Fig. 4E; unpaired two-tailed t-tests within age, n = 10–19 eyes per group; 10 w: *P* < 0.05; 36 w: *P* < 0.001), indicating that *Itga9* is required for SC development and IOP homeostasis. To assess consequences of chronic IOP elevation, we quantified RBPMS-positive RGC density at 36 weeks on flat mounts and compared genotypes within each retinal eccentricity. RGC density was significantly decreased in the distal retina of *Itga9* CKO, with no difference in central or mid-peripheral regions (Fig. 4F,G; unpaired two-tailed t-tests, n = 5–8 eyes per group; distal: *P* < 0.05; central/mid-peripheral: ns), suggesting region-specific RGC loss, phenocopying *Piezo1*^ΔEC^.

**Figure 5.**
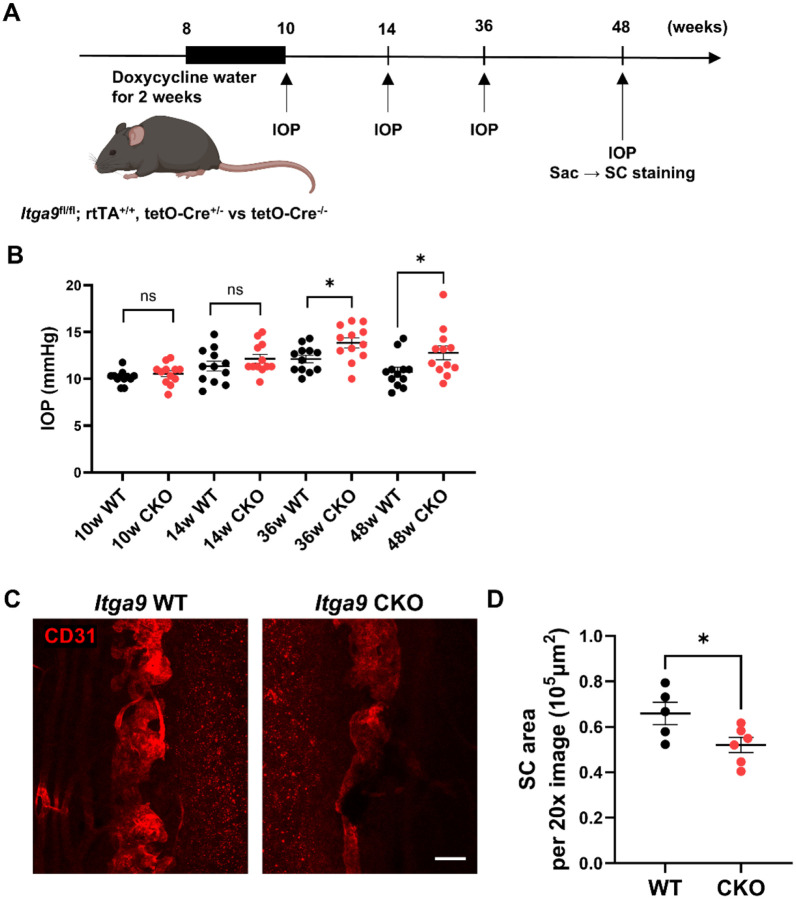
Postnatal deletion of *Itga9* leads to late-onset IOP elevation and SC narrowing. (A) Schematic of the experimental timeline for postnatal *Itga9* deletion using doxycycline-inducible tetO-Cre. Doxycycline was administered from 8 to 10 weeks of age in *Itga9*^fl/fl^; rtTA^+/+^; tetO-Cre^+/−^ (*Itga9* CKO) mice. IOP was measured at 10, 14, 36, and 48 weeks; eyes were collected at 48 weeks for SC morphometry. (B) Longitudinal IOP measurements show no difference at 10 or 14 weeks, but significantly elevated IOP in CKO mice at 36 and 48 weeks. (C) Representative SC whole-mount images from wild-type (WT) and *Itga9* CKO mice at 48 weeks stained with anti-CD31. Scale bar: 100 μm. (D) Quantification of SC area per 20× image shows a significant reduction in CKO mice compared with WT controls. Data are presented as mean ± SE. Statistical analysis was performed using two-tailed unpaired Student’s t-test. *P < 0.05, ns = not significant.

To test whether *Itga9* is also required for adult maintenance of SC, we induced deletion postnatally by administering doxycycline from 8 to 10 weeks and measured IOP at 10, 14, 36, and 48 weeks (Fig. 5A). IOP did not differ at 10 or 14 weeks but was significantly elevated at 36 and 48 weeks in *Itga9* CKO (Fig. 5B; unpaired two-tailed t-tests within time point, n = 12 mice per group; 36 w: *P* < 0.05; 48 w: *P* < 0.01). Correspondingly, SC area was reduced at 48 weeks in CKO compared with controls (Fig. 5C,D; unpaired two-tailed t-test, n = 5–6 eyes per group; *P* < 0.01), indicating that *Itga9* is also essential for long-term structural maintenance of SC and suppression of age-related IOP elevation in adulthood.

### Heterozygous deletion of *Piezo1* and *Itga9* reveals combined effects on SC narrowing and IOP elevation

**Figure 6.**
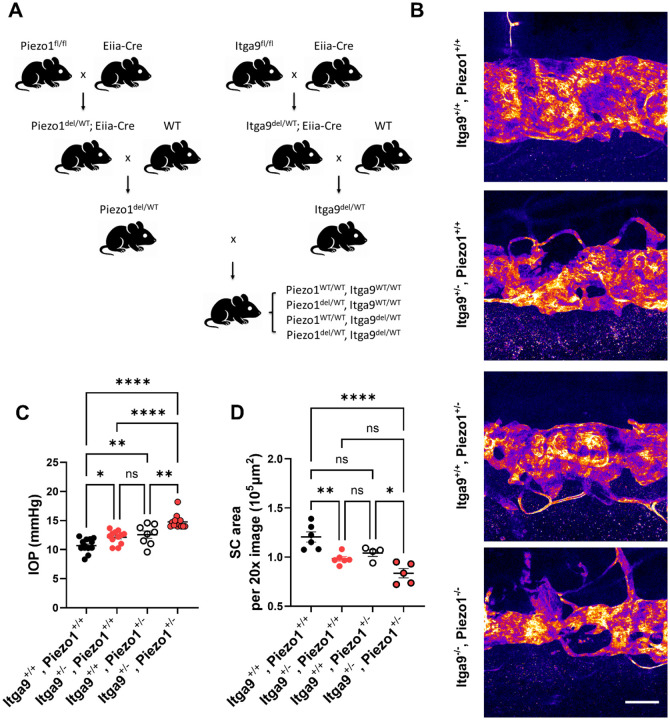
Combined heterozygous deletion of *Piezo1* and *Itga9* synergistically impairs SC morphology and elevates IOP (A) Breeding scheme for generating *Piezo1*^del/WT^; *Itga9*^del/WT^ double heterozygous mice. Germline deletion was induced by crossing *Piezo1*^fl/fl^ or *Itga9*^fl/fl^ mice with EIIa-Cre, and Cre was subsequently bred out by crossing WT mice. The resulting *Piezo1*^del/WT^ and *Itga9*^del/WT^ mice were intercrossed to generate four genotypes: *Piezo1*^WT/WT^; *Itga9*^WT/WT^, *Piezo1*^del/WT^; *Itga9*^WT/WT^, *Piezo1*^WT/WT^; *Itga9*^del/WT^, and *Piezo1*^del/WT^; *Itga9*^del/WT^. (B) Representative SC whole-mount images stained with anti-CD31 at 8 weeks of age for each genotype. (C) IOP measurements showed significant increases in both single heterozygotes versus WT, and were further elevated in *Piezo1*^del/WT^; *Itga9*^del/WT^ mice compared with either single heterozygote and WT. (D) SC area was significantly reduced in *Itga9*^del/WT^; *Piezo1*^WT/WT^, showed a trend toward reduction in *Piezo1*^del/WT^; *Itga9*^WT/WT^, and was further decreased in the double heterozygotes compared with WT and *Piezo1*^del/WT^; *Itga9*^WT/WT^ (with a trend versus *Itga9*^del/WT^; *Piezo1*^WT/WT^), consistent with a combined effect. Data are presented as mean ± SE. Statistical analysis was performed using one-way ANOVA followed by Tukey–Kramer post hoc test. *P < 0.05, **P < 0.01, ***P < 0.001, ns = not significant. Scale bar: (B) 100 μm.

We showed above that deleting *Piezo1* or *Itga9* alone reduces SC area and elevates IOP, yielding similar glaucomatous phenotypes. These observations suggested that the two genes act along a common axis in SC in vivo, and that partial loss of both might produce a combined effect. We first attempted to generate doxycycline-inducible double CKOs by crossing *Piezo1*^fl/fl^ and *Itga9*^fl/fl^ lines with rtTA;tetO-Cre, but no viable double-CKO pups of the expected genotypes were obtained—even without doxycycline—consistent with embryonic lethality from leaky Cre activity. As an alternative, we created germline-deleted heterozygous alleles (Fig. 6A): *Piezo1*^fl/fl^ or *Itga9*^fl/fl^ mice were crossed with EIIa-Cre to induce germline deletion; the resulting *Piezo1*^del/WT^ or *Itga9*^del/WT^ offspring were crossed to WT to remove EIIa-Cre; finally, *Piezo1*^del/WT^ and *Itga9*^del/WT^ carriers were intercrossed to obtain four genotypes (*Piezo1*^WT/WT^;*Itga9*^WT/WT^, *Piezo1*^del/WT^;*Itga9*^WT/WT^, *Piezo1*^WT/WT^;*Itga9*^del/WT^, *Piezo1*^del/WT^;*Itga9*^del/WT^). At 8 weeks of age, IOP differed across genotypes (Fig. 6C; one-way ANOVA with Tukey’s multiple comparisons, n = 8–15 mice per genotype): both single heterozygotes were higher than WT (Tukey-adjusted *P* = 0.0206 for *Itga9*^del/WT^;*Piezo1*^WT/WT^ and *P* = 0.0052 for *Piezo1*^del/WT^;*Itga9*^WT/WT^), and the double heterozygote was further elevated relative to each single heterozygote (*P* < 0.0001 vs *Itga9*^del/WT^;*Piezo1*^WT/WT^; *P* = 0.0121 vs *Piezo1*^del/WT^;*Itga9*^WT/WT^) and to WT (*P* < 0.0001). For SC area, *Itga9*^del/WT^;*Piezo1*^WT/WT^ was reduced versus WT (*P* = 0.0042), *Piezo1*^del/WT^;*Itga9*^WT/WT^ showed a trend (*P* = 0.0691), and the double heterozygote showed the greatest reduction (Fig. 6B,D; one-way ANOVA with Tukey, n = 4–6), significant versus WT (*P* < 0.0001) and versus *Piezo1*^del/WT^;*Itga9*^WT/WT^ (*P* = 0.0246), with a trend versus *Itga9*^del/WT^;*Piezo1*^WT/WT^ (*P* = 0.0896). Collectively, partial loss of both *Piezo1* and *Itga9* produces a combined effect—most evident for IOP—and drives the largest SC area reduction, supporting functional convergence of these genes in maintaining SC homeostasis and restraining IOP in vivo.

### The *Piezo1*–*Itga9* axis promotes outflow-dependent proliferation of SC endothelial cells

**Figure 7.**
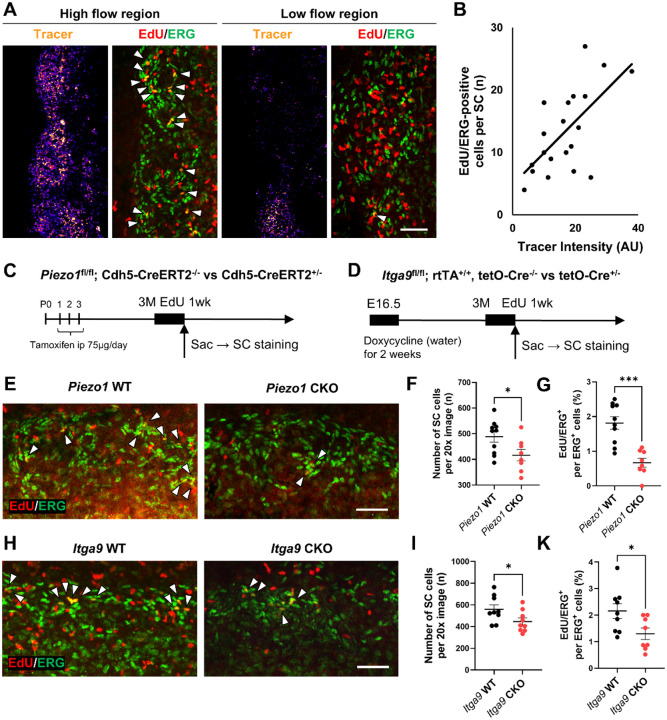
Outflow-dependent proliferation of Schlemm’s canal (SC) endothelium requires the *Piezo1–Itga9* axis. (A) SC whole-mounts after 7-day EdU labeling: a high-flow region shows more EdU^+^/ERG^+^ endothelial nuclei than a low-flow region; ERG (green), EdU (red); arrowheads mark EdU^+^/ERG^+^ cells. (B) The correlation between tracer intensity and number of EdU^+^/ERG^+^ SC endothelial cells (Pearson r = 0.643, P = 0.0022). (C) Experimental scheme for endothelial *Piezo1* deletion: *Piezo1*^fl/fl^; *Cdh5*-CreERT2^+^ vs CreERT2^−^; tamoxifen at P1–P3; EdU for 1 week at 3 months; harvest for SC staining. (D) Experimental scheme for inducible *Itga9* deletion: *Itga9*^fl/fl^; *rtTA*^+/+^; tetO-Cre^+/−^ vs tetO-Cre^−/−^; doxycycline from E16.5 for 2 weeks; EdU for 1 week at 3 months; harvest. (E) Representative SC fields from *Piezo1* WT and CKO; arrowheads indicate EdU^+^/ERG^+^ nuclei. (F) Quantification: number of SC endothelial cells per 20× image is reduced in *Piezo1* CKO. (G) Quantification: fraction of EdU^+^ nuclei within the ERG^+^ mask (EdU/ERG %) is decreased in *Piezo1* CKO. (H) Representative SC fields from *Itga9* WT and CKO. (I) Quantification: number of SC endothelial cells per 20× image is reduced in *Itga9* CKO. (K) Quantification: EdU/ERG % is decreased in *Itga9* CKO. Data are presented as mean ± SE. Statistics: two-group comparisons (F, G, I, K) used unpaired two-tailed Student’s t-test. *P < 0.05, ***P < 0.001, ns = not significant. Scale bars: (A, E, H) 100 μm.

*PIEZO1* is a mechanosensitive ion channel and is expected to function as a key mechanosensor in SC, where aqueous humor outflow provides the dominant mechanical input. In lymphatic endothelium, flow/shear activates PIEZO1 to drive endothelial expansion and proliferation [15], consistent with the lymphatic-like identity reported for SC endothelium. In addition, *Itga9* (integrin α9β1) is essential for lymphatic valve formation and maintenance at flow-defined sites [24] and regulates endothelial proliferation and migration through extracellular matrix (ECM) coupling [29]. Collectively, these studies suggest *Itga9* participates in angiogenic/organogenetic programs and could contribute to SC development and maintenance. We therefore hypothesized that shear stress generated by aqueous outflow activates the *Piezo1*–*Itga9* axis to promote SC endothelial proliferation; conversely, loss of *Piezo1* or *Itga9* would blunt flow-induced proliferation, reduce endothelial cellularity, and narrow SC—thereby further reducing flow in a pathogenic feedback loop. Aqueous outflow in SC is segmental, producing local high- and low-flow regions [6]. To examine the relationship between flow and endothelial proliferation, we injected FluoSpheres^™^ into the anterior chamber at 3 months of age and, on the same day, administered EdU (5-ethynyl-2′-deoxyuridine) in drinking water (0.2 mg/mL) continuously for 7 days before tissue harvest. Whole-mount anterior segments were prepared, and for each quadrant we quantified EdU^+^/ERG^+^ SC endothelial cells alongside integrated FluoSpheres intensity. FluoSpheres intensity and EdU^+^/ERG^+^ counts were positively correlated (Pearson *r* = 0.643, *P* = 0.0022), supporting a shear-dependent increase in local SC endothelial proliferation (Fig. 7A,B). We next tested genetic requirements. In *Piezo1*^ΔEC^ mice, both the number of SC endothelial cells and the fraction of ERG-EdU double-positive cells in the canal were reduced, indicating impaired endothelial proliferation (Fig. 7E–G). Similarly, doxycycline-inducible *Itga9* CKO (*Itga9*^fl/fl^; rtTA^+/+^; tetO-Cre^+/−^) showed decreased EdU labeling and reduced SC endothelial cellularity (Fig. 7D,H–K), phenocopying *Piezo1*^ΔEC^. Together, these results indicate that mechanical cues from aqueous outflow are sensed by PIEZO1 and transduced through ITGA9 to drive local SC endothelial proliferation and preserve canal structure. Disruption of this axis by loss of *Piezo1* or *Itga9* impairs the proliferative response, leading to reduced endothelial cellularity and canal narrowing, which in turn exacerbates outflow reduction in a deleterious feedback loop.

## Discussion

Paracrine TM-SC signaling through the ANGPT-Tie2 pathway is one of the best-known regulators of SC function and IOP homeostasis [9,10]. Here, we report that in addition to this ANGPT1-mediated paracrine pathway, a second, mechanosensitive autocrine ANGPT signaling pathway exists in the SC endothelium and regulates intraocular pressure in mouse eyes.

### PIEZO1 is a mechanosensitive regulator of the SC endothelium

Shear stress is a critical regulator of SC function, and it has been proposed that pressure-induced changes in SC lumen morphology function to amplify shear stress signals on the canal endothelium—an amplification that is lost as tissue stiffness increases with age or in glaucoma [16]. However, how SC endothelial cells detect shear and other physical stimuli and convert them to functional, IOP altering changes is poorly understood. PIEZO proteins are mechano-sensing cationic channels that play multiple roles in regulating cellular events in response to mechanical forces and fluid flow; in the conventional outflow pathway, PIEZO1 supports mechanotransduction and its pharmacologic manipulation alters outflow metrics [17]. Previous studies have linked variants in PIEZO1 to PCG in children and POAG in adults, and administration of a PIEZO1 agonist, Yoda1, reduced IOP in mice, while the inhibitor GsMTx4 significantly decreased outflow facility [17], although neither small molecule is tissue specific and previous studies did not differentiate between TM- and SC-driven effects. Here, our finding that endothelial, but not TM-specific *Piezo1* deletion led to IOP elevation suggests that inhibition of SC PIEZO1 activity was likely responsible for the facility decrease seen in previous studies with GsMTx4.

### ANGPT2 is a critical modulator of mechanosensitive signals in SC

Unlike ANGPT1, which is continuously secreted by TM and perivascular cells, ANGPT2 is stored in endothelial cells within specialized secretory vesicles called Weibel–Palade bodies (WPBs) and exocytosed in response to extracellular stimuli; ANGPT2 storage/release from WPBs is well established [30] and WPB exocytosis is Ca^2+^-dependent. We have previously reported that PIEZO1 activation by Yoda1 in lymphatic endothelial cells induces rapid ANGPT/TIE signaling [14]. Consistent with these findings, a recent preprint reported that in blood-vascular endothelial cells in vitro, PIEZO1 activation by Yoda1 or physical stimulation leads to rapid exocytosis of WPBs and release of their contents [31]. Here, we found a similar mechanism in SC, and in vivo PIEZO1 activation with Yoda1 induced rapid ANGPT2 exocytosis, visualized as loss of intracellular staining with anti-ANGPT2 antibody. Once secreted, ANGPT2 then regulates SC endothelial cells through both the classical ANGPT receptor TIE2 and a second, integrin pathway through α9β1 to induce FAK phosphorylation [13,20]. Within SC, TIE2 signaling is largely mediated by the TM-expressed ligand ANGPT1, which is highly and constitutively expressed by cells of the juxtacanalicular meshwork; *Angpt1* is required for SC development/maintenance [10], and human *TEK* variants cause PCG [9].

However, in mice, *Tek* deletion results in a more severe phenotype than loss of *Angpt1*, indicating signaling by other ligands—including *Angpt2* [10]. Prior work also shows that while *Angpt2* deletion in young mice may have minimal effects on IOP or SC morphology, older mice exhibit reduced SC size and SC-endothelial proliferation with diminished TIE2 phosphorylation [10]. In contrast to this modest effect on TIE2 signaling, in vitro, ANGPT2 knockdown almost completely ablated PIEZO1-induced FAK activation, while activation was increased by ANGPT2 stimulation—even in the absence of Yoda1. Together, these findings suggest that the PIEZO1–ANGPT2 pathway is a central regulator of integrin signaling in endothelial cells, including SC.

### Integrin α9β1 regulates SC morphology and IOP homeostasis in response to PIEZO1-induced ANGPT2 secretion

Integrins are heterodimeric cell-surface receptors that mediate interactions with the extracellular matrix and regulate adhesion, migration, cytoskeletal dynamics, and proliferation via FAK signaling. Previous studies have revealed critical roles for β-subunit proteins (e.g., ITGB1) in SC development, while multiple α-subunits—including *Itga5*, *Itga6*, and *Itga9*—are expressed in SC endothelium [7,8]. Of these, *Itga9* was especially of interest because α9β1 has established roles in lymphangiogenesis, lymphatic endothelial proliferation, and valve formation [24] and serves as a receptor for the ECM protein SVEP1 [26], which can interface with the Ang/Tie axis via TIE1 [27]. In vitro, siRNA-mediated ITGA9 knockdown ablated the effect of PIEZO1 activation on FAK phosphorylation, implicating α9β1 as the key link in the PIEZO1–ANGPT2–FAK pathway. In vivo, developmental *Itga9* deletion attenuated SC formation and produced a PCG-like phenotype, and adult deletion led to progressive IOP elevation with gradual SC reduction; a milder version of these phenotypes was seen in *Itga9* heterozygotes and was amplified by compound heterozygosity with *Piezo1*, highlighting the importance of the PIEZO1-induced pathway in outflow homeostasis.

### The PIEZO1-induced integrin signaling pathway regulates proliferation of SC endothelial cells in a flow-dependent fashion.

Although IOP is uniform throughout the anterior chamber, aqueous humor outflow through the conventional pathway is segmental, with high- and low-flow regions reflecting local differences in outflow resistance across the TM and SC inner wall; these regions shift over time, implying an actively regulated process to maintain stable IOP [16]. Our data indicate that as part of this process, PIEZO1-induced ITGA9 signaling promotes proliferation of SC endothelial cells in high-flow (high-shear) segments; loss of this pathway decreases proliferation and cellularity and reduces SC size over time. Previous studies have shown similarly reduced proliferation in *Angpt2* knockout mice [10], supporting the role of ANGPT2 within this Piezo1-mediated integrin signaling pathway.

## Conclusion

Together, our findings define a mechanosensitive autocrine ANGPT network in the SC endothelium in which shear-driven *Piezo1* activation mobilizes ANGPT2 to engage both the TIE2 arm and the α9β1–FAK arm, sustaining junctional integrity, flow-dependent endothelial proliferation, canal caliber, and IOP homeostasis. Genetic disruption of *Piezo1* or *Itga9* narrows SC and elevates IOP—with compound heterozygosity producing the largest effects—whereas reinforcing TIE2 signaling lowers IOP [32]. These results position the FLOW→PIEZO1→ANGPT2/TIE2 and ANGPT2–α9β1–FAK axis as a targetable mechanism for restoring outflow function in glaucoma.

## Materials and Methods

### Study approvals

Animal experiments were approved by the Animal Care and Use Committee at Northwestern University (Evanston, IL, USA) and comply with ARVO guidelines for care and use of vertebrate research subjects in Ophthalmology research. Animal generation and husbandry All mice were housed in the Center for Comparative Medicine at Northwestern University (Chicago, IL, USA) under standard conditions (12-hour light/dark cycle, ambient temperature 21–23 °C, 30–70% relative humidity) with ad libitum access to water and standard chow (Teklad #7912, Envigo, Indianapolis, IN, USA). *Itga9*^fl/fl^ mice were a generous gift from Dr. Livingston Van De Water (Albany Medical College), originally described in Singh et al. [33]. *Piezo1*^fl/fl^ mice (B6.Cg-*Piezo1*^tm2.1Apat^, JAX stock #029213), *Cdh5*-CreERT2 mice (Tg(*Cdh5*-cre/ERT2)1Rha, JAX, MGI:3848982), and *Rosa26*-rtTA mice (Gt(ROSA)26Sor^tm1(rtTA,EGFP)^Nagy, JAX stock #005670) were obtained from The Jackson Laboratory (Bar Harbor, ME, USA). LSL-Salsa6f reporter mice (B6(129S4)-Gt(ROSA)26Sor^tm1.1(CAG-tdTomato/GCaMP6f)^Mdcah/J, JAX stock #031968) were also obtained from The Jackson Laboratory. Inducible gene deletion *Itga9*^fl/fl^ mice were crossed with *Rosa26*-rtTA and tetO-Cre transgenic mice to generate doxycycline-inducible *Itga9* CKO mice. Pregnant dams were treated starting at embryonic day 16.5 (E16.5) by giving 0.5% (wt/vol) doxycycline-containing drinking water (with 5% sucrose), which was continued for 2 weeks to induce Cre-mediated recombination during fetal development. *Piezo1* CKO mice were generated by crossing *Piezo1*^fl/fl^ mice with *Cdh5*-CreERT2 mice. Tamoxifen (75 μg/day; Sigma-Aldrich) was administered intraperitoneally once daily from postnatal day 1 to 3 (P1–P3) to induce endothelial-specific recombination. Endothelial Ca^2+^ imaging LSL-Salsa6f mice were crossed with *Cdh5*-CreERT2 to generate *Cdh5*-CreERT2^+^; LSL-Salsa6f^+/−^ reporters. To induce reporter expression, tamoxifen (75 μg/day, i.p.) was administered for three consecutive days at 8 weeks of age. Salsa6f localizes to the cytoplasm and is excluded from nuclei, enabling ratiometric intracellular Ca^2+^ measurements based on the green/red (G/R) fluorescence ratio. Recombination site validation *Itga9* and *Piezo1* floxed lines were crossed with *Rosa26*^mTmG^ (Gt(ROSA)26Sor^tm4(ACTB-tdTomato,-EGFP)^Luo/J, JAX #007576) mice to visualize Cre activity in target tissues. Double-heterozygous experiments *Itga9*^fl/fl^ and *Piezo1*^fl/fl^ mice were crossed with EIIa-Cre (Tg(EIIa-cre)C5379Lmgd/J, JAX #003724) mice to generate germline-deleted alleles (*Itga9*^del/WT^ and *Piezo1*^del/WT^), followed by Cre transgene removal by backcrossing to wild-type C57BL/6J mice. Double-heterozygous mice were obtained by intercrossing *Piezo1*^del/WT^ and *Itga9*^del/WT^ carriers. All strains were maintained on a mixed genetic background free from RD1 and RD8 mutations. Both male and female animals were used for all experiments. Imaging was performed at 8–10 weeks of age unless otherwise indicated. Genotyping was performed by PCR using primers listed in Supplementary Table S1.

### Single-cell RNA-seq re-analysis

Public mice single-cell RNA-seq data [19] were re-analysed to compare SC endothelium and TM with respect to *Piezo1* and *Itga9* expression. Processed count matrices and the authors’ cell annotations were downloaded from the NCBI Gene Expression Omnibus (GEO Series GSE168200; BioProject PRJNA706441; SRA SRP309170). Analyses were performed in R (v4.4.2) using Seurat (v5.2.0). Cell identities followed the original annotations; all TM-related clusters reported by Thomson et al. were collapsed into a single “TM” group, and SC endothelial cluster(s) were extracted as “SC”. From this TM+SC subset, expression distributions of *Piezo1* and *Itga9* were plotted with Seurat VlnPlot. Parameters not specified here matched the defaults in Seurat and the original study.

### IOP measurements

IOP measurements were performed in awake mice between 9 and 11 AM using an iCare TonoLab rebound tonometer as previously described. Cohorts of mutant mice with littermate controls were measured at each reported timepoint. IOP values from left and right eyes were averaged to obtain values reported in the manuscript.

### Cell culture and immunostaining

Primary human dermal lymphatic endothelial cells (HDLECs; PromoCell, C-12216) were cultured in endothelial cell growth medium MV2 (PromoCell, C-22121) supplemented with the manufacturer’s supplement mix, in glass-bottomed culture plates at 37 °C in a humidified incubator with 5% CO_2_. Cells between passages 3 and 6 were used for all experiments. For pharmacological stimulation, HDLECs were treated with 2 μM Yoda1 (MilliporeSigma, SML1558) or vehicle control (DMSO) for 30 minutes prior to fixation.

### Immunofluorescence staining

Cells were fixed in 4% paraformaldehyde (PFA) for 10 minutes at room temperature, permeabilized with TBS containing 0.1% Triton X-100 for 10 minutes and blocked with TBS containing 5% donkey serum for 1 hour at room temperature. Primary and secondary antibodies were diluted in the same blocking buffer and incubated at room temperature. Nuclei were counterstained with DAPI. Fluorescence images were acquired using a Nikon A1 confocal microscope. All image processing and analysis were performed using Fiji software (ImageJ version 1.54p). Antibody information is provided in Supplementary Table S2. For colocalization analysis, Pearson’s correlation coefficient (R) was calculated between the red (ZO-1) and green (ITGA9 or ITGB1) channels using the Coloc2 plugin in Fiji with the “above threshold” setting to reduce background signal. At least 12 images (40× magnification) per group were acquired across three independent experiments for analysis.

### Western blotting

HDLECs were transfected with siRNAs using Lipofectamine RNAiMAX reagent (Thermo Fisher Scientific, 13778075) according to the manufacturer’s instructions. Silencer Select siRNAs targeting human ITGA9 (Assay IDs s7554 and s7555; Thermo Fisher Scientific, Ambion; ordered under Cat# 4392420) and Silencer Select Negative Control No. 1 siRNA (Thermo Fisher Scientific, Cat# 4390843) were used at a final concentration of 15 nM. Transfection was performed on subconfluent cultures, and cells were harvested for downstream analysis 48 hours after transfection. For Yoda1 stimulation, 2 μM Yoda1 or DMSO was added 30 minutes prior to cell lysis. Antibody information used for immunoblotting is provided in Supplementary Table S2.

### Intracameral injection and fixation

Mice were anesthetized with isoflurane (3% induction, 1.5–2% maintenance) and placed on a heated platform. FluoSpheres^™^ (20 nm, carboxylate-modified polystyrene, Thermo Fisher Scientific) were diluted in sterile PBS containing Ca^2+^/Mg^2+^ to a final concentration of 1 × 10^11^ particles/mL (~0.06% v/v). A total of 1 μL of tracer solution was sequentially loaded into a 10 μL NanoFil syringe (World Precision Instruments) and separated by a small air bubble (~0.2 μL). The syringe was fitted with a 35G beveled needle (NF35BV, WPI) and mounted on an UltraMicroPump (UMP3, WPI) connected to a MICRO-2T SMARTouch^™^ controller (WPI) and a micromanipulator. The tracer was first delivered into the anterior chamber. Yoda1 (20 μM in PBS) was injected in the same manner as described above. Eyes were collected and immersion-fixed overnight in 2% PFA at 4 °C.

### EdU assay

To label proliferating cells, EdU (5-ethynyl-2’-deoxyuridine; Carbosynth) was administered in the drinking water at a final concentration of 200 μg/mL for 7 consecutive days. EdU was prepared as a 10 mg/mL stock solution in DMSO and diluted 1:50 in sterile drinking water. The solution was replaced every 2 days and protected from light using foil-wrapped bottles. After the labeling period, mice were euthanized and the anterior segment of the eyes were carefully dissected and fixed in 2% PFA in PBS overnight at 4 °C. The next day, tissues were washed in TBS-T (3 × 5 min) and permeabilized/blocking buffer was applied for 1 hour at room temperature. The blocking buffer consisted of 5% donkey serum, 2.5% bovine serum albumin (BSA), and 0.5% Triton X-100 in TBS. EdU detection was performed using a custom copper-catalyzed azide–alkyne cycloaddition (CuAAC) reaction. A Click reaction buffer was freshly prepared containing 4 mM CuSO₄ (Acros Organics), 100 mM sodium ascorbate (Acros Organics; freshly made), and 5 μM sulfonated Alexa Fluor azide (e.g., Sulfo-Cyanine3 Azide, Lumiprobe) in TBS (pH 7.6). Tissues were incubated in this buffer for 30–60 minutes at room temperature in the dark, followed by 3 washes in PBS (10–15 min each). Sulfonated azide dyes were used to reduce non-specific background staining in whole-mount tissue. Following EdU development, tissues were again blocked for 15–30 minutes and subjected to immunofluorescence staining. Samples were incubated with primary antibodies diluted in TBS containing 1% BSA and 0.3% Triton X-100, overnight at 4 °C. After washing (3 × 15 min in TBS), tissues were incubated with species-appropriate fluorescent secondary antibodies for 1 hour at room temperature in the dark. Nuclei were counterstained with DAPI (1 μg/mL, 15 min to overnight), followed by a final wash and mounting with antifade medium. All steps involving fluorophores were carried out under light-protected conditions.

### SC and RGC immunofluorescence imaging and quantification

To evaluate SC and RGCs, whole-mounted anterior segments and retinas were subjected to immunofluorescence staining. Enucleated eyes were fixed overnight in 2% paraformaldehyde (PFA) at 4 °C. After fixation, conjunctiva and residual connective tissue were removed. A circumferential incision was made approximately 1 mm posterior to the limbus to remove the posterior segment and lens, followed by four radial incisions to prepare a petal-shaped anterior flat mount. Tissues were blocked and permeabilized for 1 hour at room temperature in TBS containing 5% donkey serum, 1% BSA, and 0.3% Triton X-100. Samples were incubated overnight at 4 °C with primary antibodies, followed by TBS-T washes and detection with species-appropriate fluorophore-conjugated secondary antibodies. Primary and secondary antibodies used are listed in Supplementary Table S2. After immunostaining, small relaxing cuts were made around the cornea to flatten the SC region. The tissue was mounted in antifade reagent with the outer scleral surface facing the coverslip. Confocal imaging was performed using a Nikon A1 microscope to acquire Z stacks of the central region of each quadrant using a 20× objective lens. Maximum-intensity projections were used for quantification, and canal area was measured in Fiji. Expression levels within SC (e.g., p-AKT, ITGA9, p-FAK) were reported as background-subtracted mean fluorescence per mm^2^ of CD31^+^ SC area.

Background subtraction was performed by subtracting the mean gray value of a same-field cell-free region of interest (ROI) from the mean gray value within the SC ROI; negative values were clipped to zero. All acquisition settings (laser power, detector gain, offset, pinhole, pixel size, and Z-step) were kept identical across groups. All image analysis was performed in a blinded fashion. For RGC analysis, retinas were flat-mounted and imaged using a Ti2 microscope (Nikon) at 20× magnification. Four images each were obtained from the proximal (0.1 mm from the optic disc), middle (0.8 mm), and distal (1.5 mm) retina in each quadrant (total of 12 fields per retina). Images were cropped to 200 × 200 μm and RGCs were manually counted. The average RGC density (cells/mm^2^) was calculated.

### Statistical analysis

Analysis of physiological, histological, and single-cell transcriptomic data was performed using GraphPad Prism 10.0.4 (GraphPad Software, San Diego, CA, USA), R version 4.4.2, or JMP version 16.0.0 (SAS Institute, Cary, NC, USA). Statistical significance was assessed using unpaired two-tailed Student’s t-test or one-way ANOVA with Tukey–Kramer’s test, as appropriate. The specific statistical test used for each dataset is indicated in the corresponding figure legend. All data are presented as mean ± standard error (SE). P values less than 0.05 were considered statistically significant and are denoted as follows: *P < 0.05, **P < 0.01, ***P < 0.001, ****P < 0.0001. All analyses were performed in a blinded fashion with respect to treatment or genotype.

## Supplementary Material

Supplement 1

Supplement 2

## Data Availability

The data that support the findings of this study are available from the corresponding author upon reasonable request. Public single-cell RNA-seq data used for re-analysis are available from the NCBI Gene Expression Omnibus (GEO) under accession GSE168200.
