# Supplementary material for: Piezo1 Activates an Autocrine Angiopoietin-2-Integrin Signaling Loop in Schlemm’s Canal to Regulate Intraocular Pressure
Source: bioRxiv. 2025 Oct 24:2025.10.24.683742. Preprint. [Version 1] doi: 10.1101/2025.10.24.683742 (PMC12633313; doi:10.1101/2025.10.24.683742)
Supplement: Supplement 1 [file media-1.pdf]

Supplementary Information

Table S1. Primer sequences used for genotyping.

Generic Cre	F/R	Sequence (5' → 3')
**Itga9 floxed allele**	forward	CCTTACAGGGCTCTAGGAAAGGGG
	reverse	AATAGTCATTGAGACTCTCCCTGG
**Piezo1 floxed allele**	forward	GCCTAGATTCACCTGGCTTC
	reverse	GCTTTAACCATTGAGCCATCT
**mTmG (GFP cassette)**	forward	GGGCACAAGCTGGAGTACAA
	reverse	GTCCATGCCGAGAGTGATCC
**Rosa26-rtTA allele**	forward	AAGGGAGCTGCAGTGGAGTA
	mutant reverse	GGCGAGTTTACGGGTTGTTA
	WT reverse	TCCGAGGCGGATCACAAGCA
**Generic Cre**	forward	GTGCAAGTTGAATAACCGGAAATGG
	reverse	AGAGTCATCCTTAGCGCCGTAAATCAAT
**Cdh5-CreERT2**	forward	ATGCAAGCTGGTGGCTGGACC
	reverse	GATCTCCACCATGCCCTCTACAC
