# Supplementary material for: Piezo1 Activates an Autocrine Angiopoietin-2-Integrin Signaling Loop in Schlemm’s Canal to Regulate Intraocular Pressure
Source: bioRxiv. 2025 Oct 24:2025.10.24.683742. Preprint. [Version 1] doi: 10.1101/2025.10.24.683742 (PMC12633313; doi:10.1101/2025.10.24.683742)
Supplement: Supplement 2 [file media-2.pdf]

Table S2. Materials used in the present study

Reagent	Company	Identifiers
Anti-PECAM-1 (CD31)	BD Biosciences	553370
Anti-ITGA9	R&D Systems	AF3827
Anti-ITGB1	R&D Systems	MAB17781
Anti-ZO-1	Invitrogen	40-2200
Anti-Phospho-FAK (Tyr397)	Invitrogen	44-625G
Anti-Phospho-TIE2	R&D Systems	AF2720
Anti-Phospho-AKT (Ser473)	Cell Signaling Technology	9271
Anti-FOXO1	Cell Signaling Technology	C29H4
Anti-RBPMS	PhosphoSolutions	1832-RBPMS
Anti-PROX1	R&D Systems	AF2727
Anti-Integrin α9 (Human)	R&D Systems	MAB4574
ERG	Abcam	AB92513
Anti-ANGPT2	Gift from Dr. Gou Young Koh	NA
